# Myeloid malignancies in cancer patients treated with poly(ADP-ribose) polymerase (PARP) inhibitors: a case series

**DOI:** 10.1038/s41408-022-00607-7

**Published:** 2022-01-25

**Authors:** Jennifer L. Oliveira, Patricia T. Greipp, Aruna Rangan, Aminah Jatoi, Phuong L. Nguyen

**Affiliations:** 1 Division of Hematopathology, Mayo Clinic, Rochester, MN USA; 2 Division of Laboratory Genetics and Genomics, Mayo Clinic, Rochester, MN USA; 3 Division of Medical Oncology, Mayo Clinic, Rochester, MN USA

**Keywords:** Leukaemia, Translational research

Dear Editor,

Poly (ADP-ribose) polymerase (PARP) inhibitors interact with DNA at single-strand breaks, prevent repair, and generate irreparable double-strand breaks that lead to tumor cell death [1]. Patients who harbor defects in homologous recombination repair, such as those with BRCA mutations, have tumors that are especially sensitive to PARP inhibitors.

However, in their meta-analysis of 28 randomized controlled trials, 18 of which were placebo-controlled, Morice et al. cited the incidence of myeloid malignancies with PARP inhibitors at 0.73% compared to 0.47% in controls (odds ratio: 2.63 (95% confidence interval: 1.13–6.14); *p* = 0.026)) [2]. This risk is small but more than doubled, even after controlling for prior platinum-based chemotherapy.

Here, we report a series of patients with PARP inhibitor-associated myeloid malignancy. Medical records were interrogated for patients prescribed a PARP inhibitor (olaparib, rucaparib, veliparib, niraparib, talazoparib) and cross-referenced against those who underwent a bone marrow (BM) biopsy with a myeloid malignancy. Two hematopathologists independently re-reviewed all available peripheral blood and BM slides and laboratory results including molecular findings from Next Generation Sequencing (NGS). In most patients, molecular testing for myeloid-associated mutations had been performed with the OncoHeme NGS panel which interrogated 42 genes recurrently mutated in myeloid neoplasms [3]. In two patients, an 11-gene panel (*CEBPA*, *DNMT3A*, *FLT3*, *IDH1*, *IDH2*, *KIT*, *KRAS*, *NPM1*, *NRAS*, *RUNX1*, and *TP53*) had been applied. Cytogenetic findings were reviewed and reported according to the 2020 International System for Human Cytogenomic Nomenclature [4].

## Cases

Between 2014 and 2020, 583 PARP inhibitor-treated patients were identified. Nine had undergone a BM biopsy that showed myelodysplastic syndrome (MDS) in four and acute myeloid leukemia (AML) in five.

## Clinical findings

All nine were women with breast and gynecologic malignancies, reflective of common indications for these agents (Table 1).

**Table 1:** Clinical features (*n* = 9).

Patient	Sex	Solid tumor diagnosis	Age at diagnosis of solid tumor (years)	AGE at diagnosis of myeloid malignancy (years)	PARP inhibitor	Duration of PARP inhibitor	Was PARP inhibitor stopped because of myeloid malignancy?	BRCA mutation?	Other cancer therapy	AGE AT DEATH OR LAST FOLLOW UP (years)	VITAL STATUS AT TIME OF THIS REPORT
1	Female	Ovarian cancer	56	63	Niraparib	4 years	Yes	Yes	carboplatin/paclitaxel	64	Alive
2	Female	Primary peritoneal cancer	51	54	Niraparib	2+ years	Yes	No	carboplatin/paclitaxel; carboplatin/doxil	55	Alive
3	Female	Fallopian tube	75	81	Olaparib	1 year	Yes	No	carboplatin/paclitaxel	81	Deceased
4	Female	Ovarian cancer	68	77	Olaparib	1+ years	Yes	No (Li-Fraumeni syndrome)	carboplatin/paclitaxel; carboplatin/docetaxel; carboplatin/doxil	78	Alive
5	Female	Breast cancer	49	56	Olaparib talazoparib	7 months	Yes	Yes	adriamycin/cytoxan/paclitaxel/carboplatin; radiation	58	Alive
6	Female	Fallopian tube cancer	65	71	Olaparib	1+ years	Yes	Yes	carboplatin/paclitaxel; carboplatin/doxil	72	Alive
7	Female	Primary peritoneal cancer	65	75	Olaparib	1.5 years	Yes	Yes	carboplatin/paclitaxel; carboplatin/gemcitabine	75	Deceased
8	Female	Fallopian tube cancer	67	68	Olaparib	9 months	Yes	Yes (MSH6 mutation)	carboplatin/paclitaxel	68	Deceased
9	Female	Ovarian cancer	62	65	Rucaparib	18 months	Yes	Yes	carboplatin/paclitaxel	66	Deceased

In two patients the cumulative duration of PARP inhibitor therapy was greater than 2 years, and all stopped the PARP inhibitor because of a myeloid neoplasm. The median time from initiation of the PARP inhibitor to diagnosis of a myeloid neoplasm was 19 months (range: 4, 56 months), and, incidentally, from initiation of chemotherapy to the myeloid neoplasm 68 months (range: 16, 121 months). Four patients are deceased with a median survival of 0.9 months (range: 0.9 to 3 months). Among those alive, the median survival was 18 months (range: 11, 35 months).

## Peripheral blood and BM findings

In all nine patients, bicytopenia or pancytopenia was observed (Table 2). The four MDS cases (patients 1–4) included one with excess blasts-1, two with ring sideroblasts and multilineage dysplasia, and one with single lineage dysplasia. The five AML cases (patients 5–9) included two with morphologic features of pure erythroid leukemia, one with acute monocytic leukemia, and two with AML not otherwise specified.

**Table 2:** Peripheral blood, bone marrow, cytogenetic and molecular genetic findings of patients diagnosed with myelodysplastic syndrome or acute myeloid leukemia.

Patient number	1	2	3	4	5	6	7	8	9
*Peripheral blood*									
Hemoglobin (g/L)	133	108	96	100	88	89	78	83	83
MCV (fL)	100.0	112.8	93.6	100.0	89.4	109.2	105.7	98.4	95.5
ANC (×10^9^/L)	1.32	0.833	4.02	2.24	1.36	0.79	1.31	0.459	0.288
Platelets (×10^9^/L)	64	210	40	20	13	38	88	19	30
Blasts (%)	0	<1	<1	1	0	<1	0	5–19	>20
*Bone marrow*									
Cellularity	Normal	Hypocellular	Hypercellular	Hypercellular	Hypercellular	Hypercellular	Hypercellular	Hypercellular	Hypercellular
Dyserythropoiesis (%)	<10	<10	11–50	<10	<10	11–50	>50	Too few	No
Ring sideroblasts (%)	5–14	0	>15	0	0	>15	<5	5–14	NA
Dysgranulopoiesis (%)	<10	No	11–50	<10	11–50	<10	<10	No	Too few
Dysmegakaryopoiesis (%)	10–50	>50	No	>50	10–50	No	10–50	>50	No
Bone marrow blasts (%)	<5	5–9	<5	<5	20	10–19	<5	>20	>20
Pathologic diagnosis	t-MN, MDS-RS-MLD	t-MN, MDS-EB1	t-MN, MDS-RS-MLD	t-MN, MDS-SLD	t-MN, acute monocytic leukemia	t-MN, pure erythroid leukemia	t-MN, pure erythroid leukemia	t-MN, AML NOS	t-MN, AML NOS
Conventional chromosome analysis	45-46,XX,-5, add(6)(p21.3), der(7)t(7;10)(q32;q11.2),-9,-10,-12, add(21)(p11.1), +2-4mar[cp4]/ 46,XX[16]	45,XX,inv(3)(q2q26.2),-7[5/44, sl,dic(18;21) (p11.1;p11.1)[4]/ 46,XX[11]	44-46,XX,-4, add(5)(q31),+8,add(8)(p11.2), -10, inv(14)(q22q32),add(16)(q12), add(16)(q12),-18,der(19)t(18;19)(q11.2;p13.1),-20, +2-4mar[cp13]/ 45-46,idem,-8,+i(18)(q10),-der(19)t(18;19)(q11.2;p13.1),+idic(22)(q10), +2-3mar[cp6]/ 46,XX[1]	45,XX, der(17;20)(q10; p10)[1]/46,sl, del(5)(q22q35), del(7)(q22),+8[8]/46,XX[3]	46,XX t(9;11)(p22;q23)[11]/ 46,XX,idem, del(6)(p23)[7]/46,XX[2]	40-44,XX,-5,der(6;17)(q10;q10),inv(6)(p23q13),t(12;21)(q13;q22), add(13)(q12),dic(14;?)(p13;?), −16,−18,−19, add(22)(q13), +0-1mar[cp19]/ 46,XX[1]	44-47,X, add(X)(p22.3),5, add(5)(p13), der(5;7)(p10;q10), add(7)(q32), der(7;19)(q10;q10),+8,+add(9)(q13), der(16)add(16) (p11.2)add(16)(q22), -19,+0-4mar[cp14]/46,XX[6]	42,XX,del(5)(q15q33),-7, add(10)(p11.2),add(10)(q22),11,add(11)(q23, add(12)(p11.2), der(14;17)(q10;q10),-16[20]	69-74,XX,+1, add(1)(q21),+2,+4,+5,+6,+6,+7, add(7)(q11.2),+8,+8,+9,+10, +11,+12,+13,+13,+14,+15,+17, +17,+18,+18,+19,+20,+20,+21, +22, +0-2mar[cp19]/ 46,XX[1]
FISH	Not done	Not done	^a^80% = 5q-, 20q-, 9% = +8	31% = TP53- (only probe)	^b^88.6% = MLLT3/MLL(KMT2A) fusion	^b^85% = 5q-, TP53- (partial), 6p-, -16, RUNX1×3, 9.5% = MLLx3 intact, 5% = RPN1 and MECOM x3	Not done	^b^35% = 5q-, -7, TP53-, NORMAL MLL(KMT2A)	^b^91% = MLL(KMT2A) amplification, 85% = near tetraploid
Next generation sequencing	Double TP53 mutations Chr17(GRCh37):g.7578394T>C; NM_000546.4(TP53):c.536A>G; p.His179Arg (12%) Chr17(GRCh37):g.7579331G>C; NM_000546.4(TP53):c.356C>G; p.Ala119Gly (11%)	1. DNMT3A: Chr2(GRCh37):g.25457243G>A; NM_022552.4(DNMT3A):c.2644C>T; p.Arg882Cys (20%) 2. GATA1: ChrX(GRCh37):g.48649565C>T; NM_002049.3(GATA1):c.49C>T; p.Gln17* (16%) 3. RUNX1: Chr21(GRCh37):g.36252853_36252868dup; NM_001754.4(RUNX1):c.494_508+1dup; p.? (10%)	Double TP53 mutations Chr17(GRCh37):g.7578457C>T; NM_000546.4(TP53):c.473G>A; p.Arg158His (45%) Chr17(GRCh37):g.7578403C>T; NM_000546.4(TP53):c.527G>A; p.Cys176Tyr (44%)	1. DNMT3A: Chr2(GRCh37):g.25468170del; NM_022552.4(DNMT3A):c.1506del; p.Thr503Profs*148 (41%) 2. TP53: Chr17(GRCh37):g.7577106G>A; NM_000546.4(TP53):c.832C>T; p.Pro278Ser (46%)	No pathogenic mutations detected	TP53: Chr17(GRCh37):g.7578190T>C; NM_000546.4(TP53):c.659A>G; p.Tyr220Cys (69%).	Not done	^c^TP53: Chr17(GRCh37):g.7579573del; NM_000546.4(TP53):c.114del; p.Ala39Glnfs*5 (23%)	^c^TP53: Chr17(GRCh37):g.7578280G>A; NM_000546.4(TP53):c.569C>T; p.Pro190Leu (97%)

*MCV* mean corpuscular volume, *ANC* absolute neutrophil count, *t-MN* therapy-related myeloid neoplasm, *MDS-RS-MLD* myelodysplastic syndrome with ring sideroblasts and multilineage dysplasia, *MDS-EB1* myelodysplastic syndrome with excess blasts-1, *MDS-SLD* myelodysplastic syndrome with single lineage dysplasia, *AML NOS* acute myeloid leukemia, not otherwise specified.

^a^MDS FISH Panel: inversion of chromosome 3/t(3;3) (RPN1/MECOM); -5/5q-, -7/7q-, trisomy 8, -17/17p-, and 20q-.

^b^AML FISH Panel: inversion of chromosome 3/t(3;3) (RPN1/MECOM); -5/5q-, -7/7q-, 13q-, -17/17p-, 20q-, trisomy 8, rearrangements of *MLL(KMT2A), NUP98*, and several translocations: t(6;9)(*DEK*;*CAN*), t(8;21)(*RUNX1T1*;*RUNX1*), t(15;17)(*PML*;*RARA*), t(8;16)(*KAT6A*;*CREBBP*), t(9;22)(*ABL1*;*BCR*), and t(3;5)(*MLF1*;*NPM1*).

^c^For patients 8 and 9, a smaller 11-gene panel was interrogated.

The BM was hypercellular in seven cases, normocellular in one MDS case, and hypocellular in one MDS case. Dysmegakaryopoiesis was observed in three of four MDS cases (Table 2) and three of the five AML cases. Abnormally small megakaryocytes with hypolobate nuclei were the predominant morphology. Clear-cut dysgranulopoiesis was less common, observed in only two cases.

Outside of the two pure erythroid leukemia cases (patients 6 and 7), unequivocal dyserythropoiesis was seen in one case. In eight cases in which an iron stain had been performed, ring sideroblasts were detected in two MDS and three AML cases (Table 2).

## Cytogenetics

Conventional chromosome analysis showed that all except patient 5 had a complex karyotype (Table 2). Except for patients 4 and 9, six of eight patients with a complex karyotype also met the definition of a monosomal karyotype, as defined by Kayser et al [5]. Among all nine patients, the most common abnormalities were -5 or a structural abnormality that resulted in 5q- and -7 or structural abnormalities resulting in 7q-. Loss of 17p was observed in three patients (patients 4, 6, 8), all of which represented biallelic inactivation of *TP53* when investigated further by fluorescence in situ hybridization (FISH) (Table 2).

## Molecular genetics

Mutation profiling by a 42-gene NGS panel was performed in two of five AML and in all four MDS cases; in two AML cases (patients 8, 9), a focused 11-gene panel was employed. Pathogenic alterations involving the *TP53* gene were detected in three of four MDS patients and in three of four AML patients (Table 2). Despite ring sideroblasts in patients 1, 3, 6, and 7, no pathogenic alterations of the *SF3B1* gene were identified. For patient 8 in whom ring sideroblasts were detected, the smaller 11-gene NGS panel did not include *SF3B1*.

In all patients with *TP53* mutations, we observed double mutations (patients 1 and 3), a VAF consistent with loss of heterozygosity (patient 9), or biallelic inactivation by FISH analysis (patients 4, 6, 8). In five of six patients (patients 1, 3, 6, 8, 9), *TP53* was the only gene affected. In patient 4, a concurrent pathogenic *DNMT3A* gene alteration was identified. Most *TP53* mutations were missense variants in the DNA binding domain with one frameshift variant in the transactivating domain.

To our knowledge, this is the first comprehensive report of the pathologic and genetic characteristics of myeloid malignancies in patients treated with PARP inhibitors. Two findings are especially striking: 1) complex karyotypes with frequent involvement of chromosomes 5 and 7 with a prevalence of a monosomal karyotype and 2) the frequent occurrence of pathogenic *TP53* mutations, with *TP53* often the sole gene affected either as biallelic inactivation or double mutations. Although dysmegakaryopoiesis was frequently observed, we observed no unique morphologic features in this series. The NGS panel employed for this study did not include *PPM1D*; otherwise, our observations corroborate the molecular findings reported by Martin and others [6].

Complex karyotypes, frequent *TP53* mutations [7, 8] and disconnect between ring sideroblasts and *SF3B1* mutations [9, 10] distinguish PARP inhibitor-associated myeloid neoplasms from *de novo* neoplasms [11, 12]. These characteristics seem directly tied to the mechanism of action: PARP inhibitors prevent the repair of DNA single-stranded breaks and thereby give rise to double stranded breaks, so-called synthetic lethality. When these drugs cause major disruptions in genomic stability—often in patients who have *BRCA* mutations and others prone to such genomic instability—it is not surprising that the myeloid malignancies diagnosed after exposure to a PARP inhibitor demonstrate chromosomal instability and frequent alterations selectively involving the DNA-damage response gene *TP53*.

Although it is less clear whether PARP inhibitor-associated myeloid neoplasms differ from other therapy-related myeloid neoplasms, three points suggest differences. First, unlike studies of therapy-related myeloid neoplasms in which a median of three gene mutations were observed [11], here, *TP53* gene alterations were the only mutated gene in most cases. Second, *TP53* gene mutations in 75% of these patients are higher than the 30–40% frequency reported in other therapy-related myeloid neoplasms [11, 12]. Moreover, through biallelic inactivation, loss of heterozygosity, or double *TP53* mutations suggestive of biallelic inactivation, the *TP53* gene appears at higher risk for inactivation with PARP inhibitors. Although the small number of patients in this series precludes a definitive determination, our findings raise the question of a more central role of *TP53* gene alterations in PARP inhibitor-associated myeloid neoplasms, particularly in these types of patients, some of whom harbor *BRCA* mutations or other underlying defect(s) in DNA repair mechanisms. Third, in contrast to other agents, such as alkylators, which lead to the development of myeloid malignancies within 5–10 years, PARP inhibitors appear to lead to a relatively rapid development of myeloid neoplasms, as suggested by a cumulative exposure to PARP inhibitors of under 5 years and continued use of PARP inhibitor up until the diagnosis of a myeloid neoplasm. These observations suggest that PARP inhibitor-associated myeloid neoplasms merit attention as a specific entity of therapy-related myeloid neoplasms.

This case series has limitations. First, without information on *TP53* alterations as clonal hematopoiesis of indeterminate potential (CHIP) mutations prior to PARP inhibitor exposure, we cannot exclude expansion of pre-existing CHIP mutations as a contributor to myeloid neoplasms following PARP inhibitors [13]. Only a longitudinal study could assess whether CHIP or other gene alterations predispose to these myeloid neoplasms. Second, the NGS panels employed were geared towards myeloid cancers with only a limited number of cancer-predisposing genes such as *DDX41* and *PTPN11* interrogated but not others such as *CHEK2 and RTEL1*. Given recent observations of a high likelihood of an underlying cancer predisposition germline variant among patients with two or more cancers [14], a more comprehensive germline and somatic genetic analysis beyond *BRCA1/2* mutation testing might be of value in identifying patients at risk for PARP inhibitor-related myeloid neoplasms. Similarly, we cannot exclude delayed synergistic interactions between PARP inhibitors and prior chemotherapy as the cause of myeloid neoplasms, particularly as others have suggested such therapeutic synergy. Third, 6 patients rapidly transitioned to supportive care alone and 3 had only short follow up after chemotherapy for a myeloid malignancy. Further outcome data would be of value. Fourth, this series generated only nine patients, but the study by Morice and others reported 21 of 4533 patients. Thus, these nine patients -- who underwent in-depth clinical, morphologic, cytogenetic, and molecular genetic assessment – provide instructive findings.
